# Acute Treatment Regimens for Migraine in the ED

**DOI:** 10.15844/pedneurbriefs-29-3-6

**Published:** 2015-03

**Authors:** J. Gordon Millichap

**Affiliations:** 1 Division of Neurology, Ann & Robert H. Lurie Children's Hospital of Chicago, Chicago, IL; 2 Departments of Pediatrics and Neurology, Northwestern University Feinberg School of Medicine, Chicago, IL

**Keywords:** Emergency, Headache, Migraine, Therapeutics

## Abstract

Researchers at Children's Hospital, Boston, studied the comparative effectiveness of acute medication regimens for the prevention of ED visits with migraine.

Researchers at Children's Hospital, Boston, studied the comparative effectiveness of acute medication regimens for the prevention of ED visits with migraine. Children aged 7 to 18 years with a principal diagnosis of migraine headache were evaluated retrospectively using data from 35 pediatric EDs (2009-2012). The primary outcome was a revisit to the ED within 3 days. Of 32,124 children identified with migraine, 27,317 (85%) were discharged and 5.5% had a return ED visit within 3 days. Only 1 in 18 children with acute migraine required a revisit to the ED within 3 days.

At the index visit, the most common medications included nonopioid analgesics (66%), dopamine anatagonists (50%), diphenhydramine (33%), and ondansetron (21%). Triptans and opiate medications were used infrequently (3% each). Children receiving metoclopramide had a 31% increased odds for an ED revisit within 3 days compared with prochlorperazine. Diphenhydramine with dopamine antagonists was associated with 27% increased odds of an ED revisit compared with dopamine antagonists alone. Prochlorperazine is superior to metoclopramide in preventing a revisit, and diphenhydramine is associated with increased rates of return visit. [1]

COMMENTARY. A review of symptomatic treatment of migraine in children in the Netherlands found a total of 10 trials with a total of 1575 patients. Acetaminophen, ibuprofen, and nasal-spray sumatriptan were all effective compared to placebo [2]. In a study that included 14 trials (only 1 in the ED), ibuprofen and acetaminophen were more effective than placebo, whereas the efficacy of intranasal sumatriptan was unclear [3]. In a current Canadian Headache Society systematic review of treatment of migraine pain in adults in emergency settings, prochlorperazine is strongly recommended whereas the use of several compounds, including acetaminophen and sodium valproate, is not recommended [4].

Inadequate acute treatment of migraine episodes is associated with an increased risk of new-onset chronic migraine over the course of 1 year [5]. Among 5,681 patients with episodic migraine in 2006, 3.1% progressed to chronic migraine in 2007. In the group with maximum treatment efficacy of acute migraine, only 1.9% developed chronic migraine, whereas among those with very poor treatment efficacy, 6.8% developed chronic migraine. Further studies of the treatment of acute migraine are indicated.
